# D-dimer level significance for deep vein thrombosis screening in the third trimester: a retrospective study

**DOI:** 10.1186/s12884-021-04353-9

**Published:** 2022-01-08

**Authors:** Keisuke Miyamoto, Hiroaki Komatsu, Masayo Okawa, Yuki Iida, Daiken Osaku, Yukihiro Azuma, Takako Tsuneto, Takashi Harada, Fuminori Taniguchi, Tasuku Harada

**Affiliations:** Department of Obstetrics and Gynecology, Tottori University School of Medicine, 36-1 Nishicho, 683-8504 Tottori Prefecture Yonago, Japan

**Keywords:** D-dimer, Deep vein thrombosis, Pregnancy, Compression ultrasonography

## Abstract

**Background:**

Venous thromboembolism often develops after surgery and childbirth, resulting in death in some cases. Although early deep vein thrombosis (DVT) detection can predict pulmonary thromboembolism, there is no early screening method for DVT in pregnant women. Lack of consensus regarding significance or setting and cut-off value interpretation of D-dimer levels further impedes venous thromboembolism screening in pregnant women.

This study aimed to examine the utility of third-trimester serum D-dimer levels as a screening test for DVT during pregnancy and to determine the frequency of asymptomatic DVT using lower-limb compression ultrasonography.

**Methods:**

This single-center retrospective study included 497 pregnant women who underwent elective cesarean section at term in our hospital between January 2013 and December 2019. Serum D-dimer levels were preoperatively measured at 32–37 weeks’ gestation. The presence or absence of DVT in patients with serum D-dimer levels ≥ 3.0 µg/ml, the cut-off value, was examined using compression ultrasonography. In all patients, the presence or absence of clinical venous thrombosis (symptoms such as lower-limb pain, swelling, and heat sensation) was examined within 4 postoperative weeks.

The Royal College of Obstetricians and Gynecologists Guideline 2015 was referred to determine risk factors for the onset of venous thrombosis during pregnancy. Among those, we examined the risk factors for DVT that result in high D-dimer levels during pregnancy.

**Results:**

The median age and body mass index were 35 (20–47) years and 21.2 (16.4–41.1) kg/m^2^, respectively. Further, the median gestational age and D-dimer levels were 37 weeks and 2.1 (0.2–16.0) µg/ml, respectively. Compression ultrasonography was performed on 135 (26.5%) patients with a D-dimer level ≥ 3.0 µg/ml, with none of the patients showing DVT. All patients were followed up for 4 postoperative weeks, with none presenting with venous thromboembolism. Multivariate analysis showed that hypertensive disorders of pregnancy are an independent risk factor for venous thromboembolism that causes high D-dimer levels (odds ratio: 2.48, 95% confidence interval: 1.05–6.50, *P* = 0.04).

**Conclusion:**

There may be low utility in screening for DVT using D-dimer levels in the third trimester. Further, prepartum asymptomatic DVT has a low frequency, indicating the low utility of compression ultrasonography.

**Trial registration:**

Institutional Review Board of Tottori University Hospital (IRB no. 20A149).

## Background

Prevention of venous thromboembolism (VTE) is critical since it often develops after surgery and childbirth, resulting in death in some cases. Perioperative VTE prevention is generally managed based on risk factors, including the degree of surgical invasion, age, and obesity [1, 2]. The three VTE components include venous stasis, vascular endothelial damage, and hypercoagulability (Virchow’s triad) [3]. Given that pregnancy has all three components, VTE frequency is 5.5 ± 6 times higher in pregnant women than in general women of childbearing age [4, 5].

In the United Kingdom and the United States, the VTE frequency in pregnant women is estimated to be 50–200 events per 100,000 pregnancies [6]. In Japan, VTE-related deaths account for approximately 7% of all maternal deaths, with its main cause being pulmonary thromboembolism (PTE) [7]. However, PTE rarely develops independently and is often caused by deep vein thrombosis (DVT) [2]. Although early DVT detection can predict PTE, there remains no early screening method for DVT in pregnant women.

Generally, serum D-dimer levels and imaging tests, including contrast-enhanced computed tomography (CT) examination and lower-limb compression ultrasonography (CUS), are recommended as screening methods for VTE [8]. However, contrast-enhanced CT examination cannot be easily performed on pregnant women, given the effects of radiation exposure and contrast medium on the fetus. Moreover, performing CUS on all pregnant women is challenging, given the heavy burden on laboratory technicians. D-dimer levels are often increased during pregnancy. The lack of consensus regarding the significance of the measurement of D-dimer levels as well as the setting and interpretation of its cut-off values, further impedes VTE screening in pregnant women.

DVT screening is crucial since the VTE risk after cesarean section is 3.7-fold higher than that after vaginal delivery [9]. Previously, we defined patients with D-dimer levels ≥ 10 µg/ml on the first day after the cesarean section as DVT high-risk patients; further, we found that enoxaparin administration from the day after surgery could effectively prevent DVT [10]. However, the lack of DVT screening, including preoperative D-dimer levels and CUS, was observed as a limitation. Only a few studies have investigated asymptomatic DVT screening; additionally, the frequency of preoperative asymptomatic DVT remains unclear, with the frequency of asymptomatic DVT after cesarean section being reported to be 3.9% [11]. We aimed to investigate the relationship between D-dimer levels measured in the third trimester and perioperative VTE. Further, we aimed to determine the frequency of preoperative asymptomatic DVT and risk factors causing high D-dimer levels.

## Methods

This single-center retrospective study was conducted at Tottori University Hospital. We included 497 patients who underwent elective cesarean section at 37–38 weeks’ gestation from January 2013 to December 2019. Serum D-dimer levels were measured at 32–37 weeks’ gestation as a preoperative DVT screening test. The cut-off value for serum D-dimer levels was set at 3.0 µg/ml, which was twice the 95^th^ percentile value in the third trimester of approximately 1.5 µg/ml [12]. Further, we examined for the presence or absence of DVT in patients with D-dimer levels ≥ 3.0 µg/ml using lower-limb CUS. Patients with D-dimer levels < 3.0 µg/ml were followed up, with a focus on the presence or absence of clinical venous thrombosis (with symptoms such as lower-limb pain, swelling, and heat sensation). We used the latex immunoassay method to measure D-dimer levels.

**Fig. 1:**
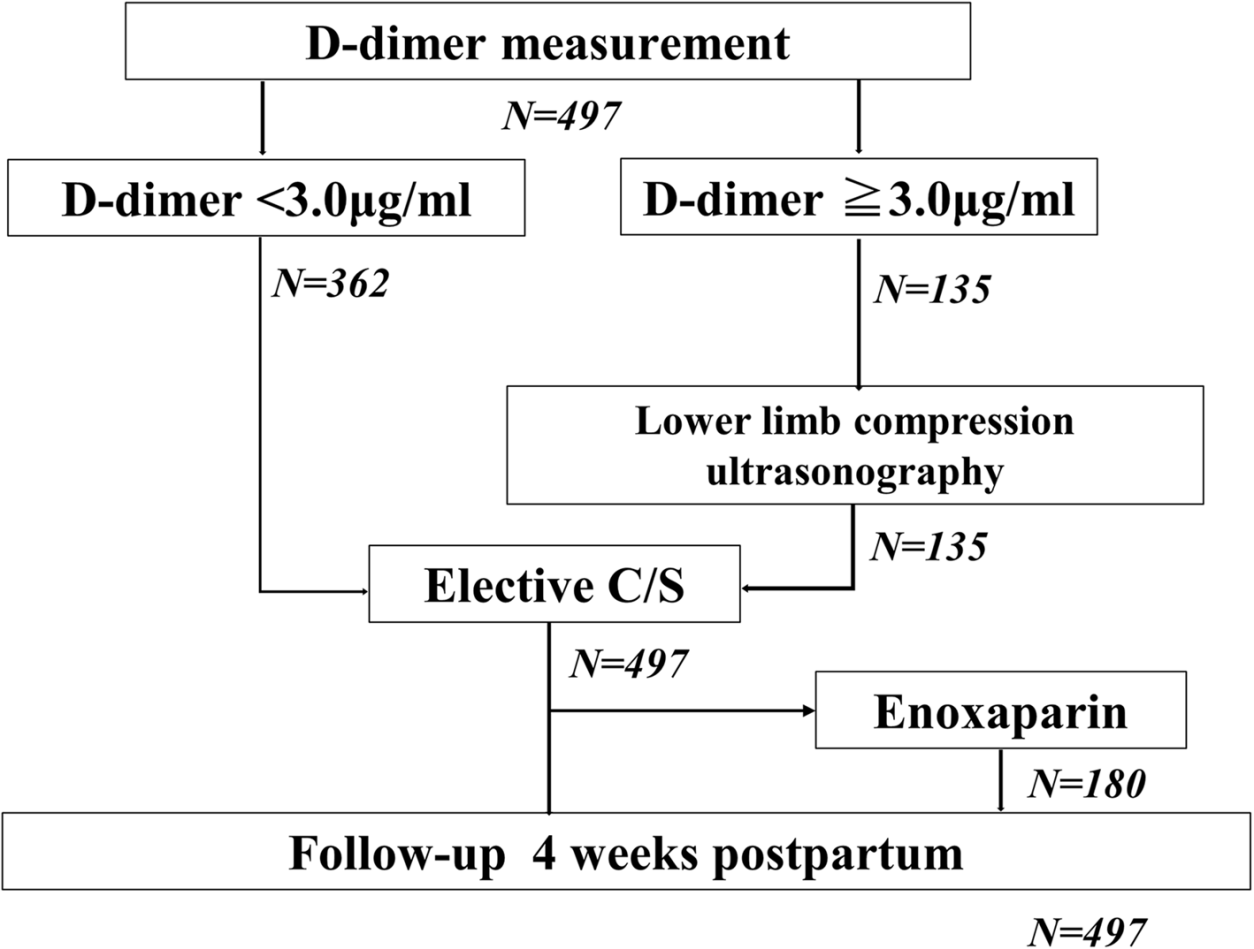
Flowchart showing screening for DVT in our institution. D-dimer levels measured in the third trimester before C/S. C/S, cesarean section; DVT, deep vein thrombosis

Eligible participants were patients managed at our hospital from mid-pregnancy to postpartum examination. In order to examine the significance of the D-dimer value as a preoperative DVT screening, pregnant women aged 32–37 weeks’ gestation, which is the timing for general preoperative examination, were included. Patients who underwent emergency cesarean section were excluded because there was no time to measure D-dimer and to perform lower-limb CUS preoperatively. Patient information, including clinical and medical history, was collected from the medical records during the first medical examination. Additionally, we examined for the presence or absence of clinical venous thrombosis within 4 postoperative weeks (Fig. 1).

The eligibility criteria for enoxaparin administration for postoperative DVT prevention included age ≥ 35 years, body mass index (BMI) ≥ 27 kg/m^2^, bedrest of ≥ 2 weeks, and thrombophilia (antiphospholipid antibody syndrome and AT-III/protein C or protein S deficiency). Additionally, enoxaparin was administered for 7 days starting from postoperative day 1, regardless of the preoperative D-dimer levels. As our treatment policy, we ensured that the patient got out of bed on postoperative day 1.

The study endpoints were the serum D-dimer levels in the third trimester, frequency of asymptomatic DVT determined through prepartum CUS, presence/absence of VTE risk factors causing high D-dimer levels during pregnancy, and presence/absence of clinical DVT within 4 postoperative weeks. VTE risk factors were determined based on the Royal College of Obstetricians and Gynecologists Guideline 2015. The high-risk factor was a history of experiencing VTE ≥ 2 times. Medium-risk factors included a history of thrombophilia; thrombosis; cardiopulmonary disease; connective tissue diseases, including systemic lupus erythematosus; malignant tumor; inflammatory bowel disease; nephrotic syndrome; and paralysis. Low-risk factors included age ≥ 35 years at pregnancy; BMI ≥ 25 kg/m^2^; and a history of hospitalization during pregnancy (≥ 14 days), multiple pregnancies, hypertensive disorders of pregnancy (HDP), thrombophilia, and smoking. HDP included preeclampsia, gestational hypertension, superimposed preeclampsia, and chronic hypertension.

The Mann–Whitney U-test and Fisher’s exact test were used to investigate the significance of differences. Moreover, we performed multivariate analysis to fit a Cox proportional hazards model. Statistical significance was set at *P* < 0.05. All statistical analyses were performed using GraphPad Prism 8.3 software (GraphPad Software, Inc., La Jolla, CA, USA).

This study was approved by the Institutional Review Board of Tottori University Hospital (IRB no. 20A149). All patients provided written informed consent following the institutional guidelines.

## Results

**Fig. 2:**
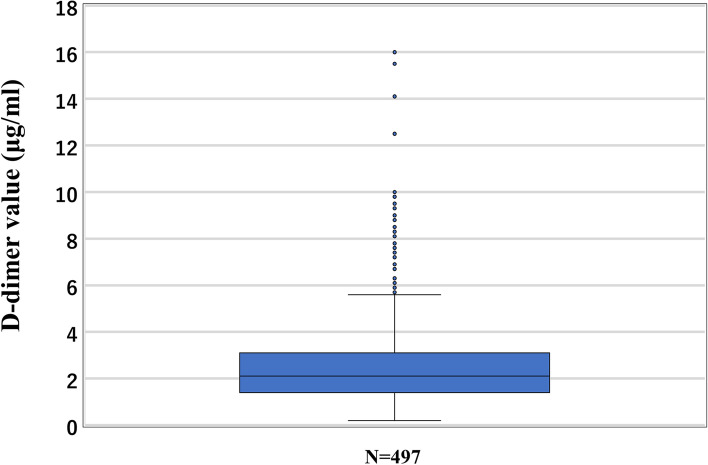
The median D-dimer level in the third-trimester before C/S was 2.1 μg/ml. C/S, cesarean section

**Fig. 3:**
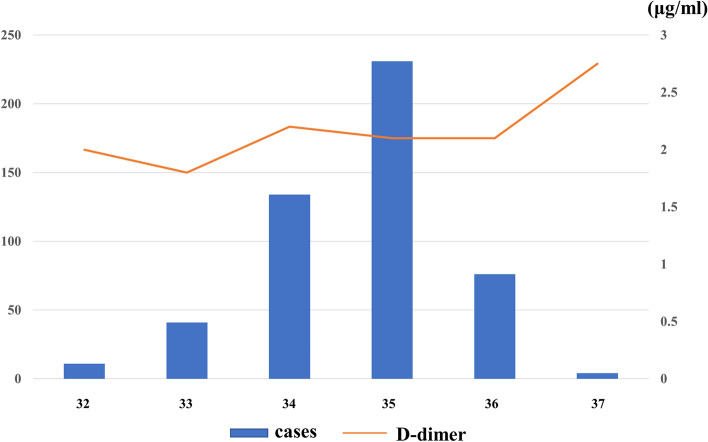
In most cases, D-dimer levels were measured at 35 weeks; further, the median D-dimer value tended to increase with each week

Table 1 presents the patients’ background characteristics. The median age and BMI at the time of D-dimer measurement were 35 (20–47) years and 21.2 (16.4–41.1) kg/m^2^, respectively. The median gestational age at delivery and D-dimer measurements were 37 and 35 weeks, respectively; further, the median D-dimer level was 2.1 (0.2–16.0) µg/ml (Figs. 2 and 3). There was no difference in the age and BMI between patients divided based on a D-dimer cut-off value of 3.0 µg/ml. CUS was performed on 135 (26.5%) patients with a D-dimer level ≥ 3.0 µg/ml, with none showing DVT. None of the patients presented with clinical VTE within 4 postoperative weeks. Enoxaparin was administered to 180 (36%) patients.

**Table 1:** Patient characteristics and outcomes

	*N* = 497	D-dimer level ≥ 3.0	D-dimer level < 3.0	***P***
		*N* = 135	*N* = 362	
Age (y)	35	35	35	0.91
	(20–47)	(21–46)	(20–47)	
BMI (kg/m^2^)	21.2	20.9	22.5	0.04
	(16.4–41.1)	(16.5–41.1)	(16.4–39.7)	
D-dimer (μg/ml)	2.1	4.6	1.8	< 0.001
	(0.2–16.0)	(3.0–16.0)	(0.2–2.9)	
Enoxaparin [%]	180[36]	45[33]	135[37]	
DVT (asymptomatic)	-	0	-	
Clinical VTE, N	0	0	0	

There was no asymptomatic DVT before C/S

*BMI* body mass index, *DVT* deep vein thrombosis, *C/S* cesarean section, *VTE* venous thromboembolism

Data are presented as median (interquartile range), N[%] or N. Comparisons were performed using the Mann–Whitney U test

**Fig. 4:**
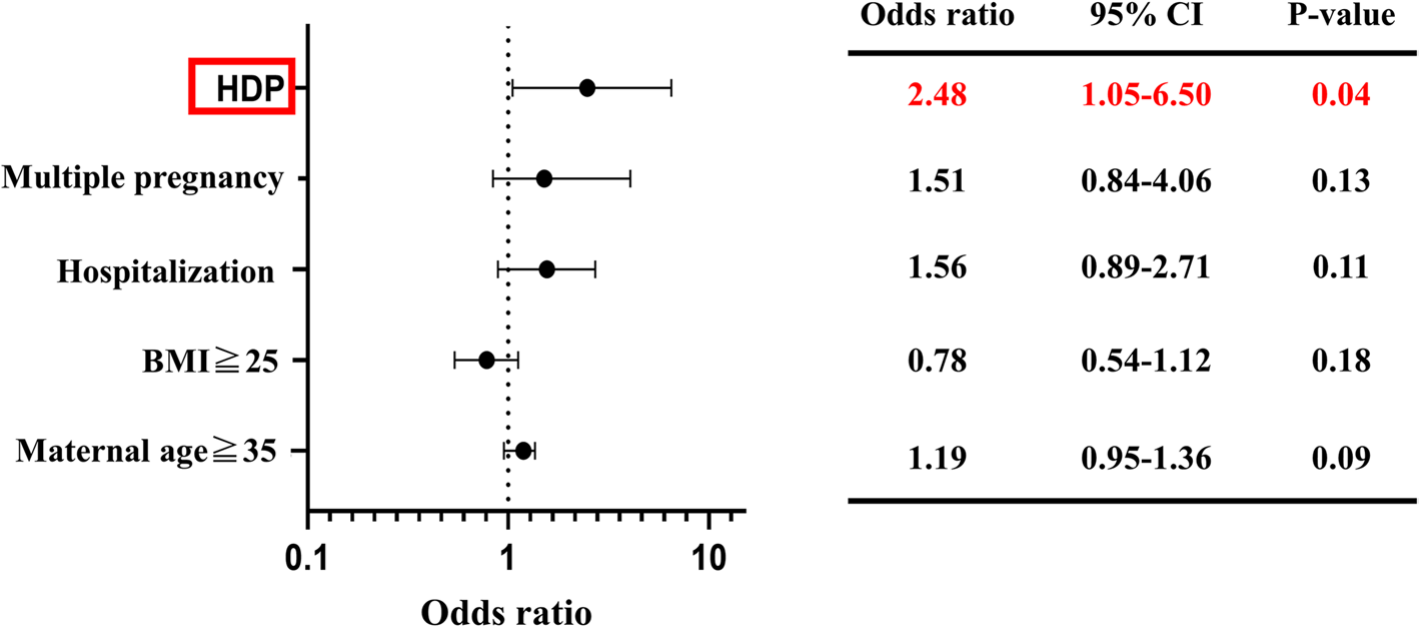
Multivariate analysis of risk factors for DVT associated with D-dimer levels of ≥ 2.1 μg/ml. HDP was an independent risk factor associated with high D-dimer levels. BMI, body mass index; CI, confidence interval; DVT, deep vein thrombosis; HDP, hypertensive disorders of pregnancy

Table 2 lists the patients with VTE risk factors. As regards the low-risk factors, 257 (51.7%) patients were ≥ 35 years old at pregnancy, 70 (14.0%) had BMI ≥ 25 kg/m^2^ at pregnancy, 113 (22.7%) had a history of hospitalization during pregnancy (≥ 14 days), 53 (10.6%) had a history of multiple pregnancies, 28 (5.6%) had a history of HDP, and 2 (0.4%) had a history of smoking. Regarding medium-risk factors, 13 (2.6%) patients had thrombophilia; 3 (0.6%) patients had connective tissue diseases, including systemic lupus erythematosus; and 2 patients (0.4%) had cardiopulmonary disease, malignant tumor, and paralysis. Notably, none of the patients presented with high-risk factors. To identify risk factors that cause D-dimer levels > 2.1 µg/ml (median value), we performed a multivariate analysis of the following low-risk factors for VTE that showed a particularly high frequency: age ≥ 35 years; BMI ≥ 25 kg/m^2^; and history of hospitalization during pregnancy, multiple pregnancies, and HDP. We identified HDP as an independent risk factor for high D-dimer levels (odds ratio: 2.48; 95% confidence interval: 1.05–6.50 (Fig. 4).

**Table 2:** Risk factors for VTE and D-dimer levels

Risk		*N* = 497(%)	D-dimer ≧3.0	D-dimer < 3.0
			*N* = 135(%)	*N* = 362(%)
Low	Age ≧35 years	257(51)	74(54)	183(50)
	BMI ≧25.0 kg/m^2^	70(14)	12(8)	58(16)
	Hospitalization ≧14 days	113(22)	46(34)	67(18)
	Multiple pregnancy	53(10)	27(20)	26(7)
	HDP	28(5)	10(7)	18(4)
	Smoker	2(0.4)	0	2(0.5)
Moderate	Thrombophilia	13(2)	5(3)	8(2)
	VTE history = 1 time	0	0	0
	Cardiopulmonary disease	2(0.4)	0	2(0.5)
	Inflammatory bowel disease	0	0	0
	Connective tissue disease	3(0.6)	3(2)	0
	Malignant tumor	2(0.4)	0	2(0.5)
	Paralysis	2(0.4)	0	2(0.5)
	Nephrotic syndrome	0	0	0
High	VTE history ≧2 times	0	0	0

Risk factors for VTE were extracted with reference to the Royal College of Obstetricians and Gynecologists Guideline 2015

*VTE* venous thromboembolism, *HDP* hypertensive disorders of pregnancy, *BMI* body mass index

## Discussion

### Principal findings

As preoperative DVT screening, we measured serum D-dimer levels in the third trimester in pregnant women scheduled for elective cesarean section. CUS revealed no DVT in pregnant women with D-dimer levels of ≥ 3 µg/ml; further, none of the patients presented with clinical VTE within 4 postoperative weeks. Furthermore, HDP was identified as a risk factor for VTE that caused high D-dimer levels.

### Results in the context of what is known

D-dimer levels increase during pregnancy, which could involve continuous functioning of the coagulation/fibrinolytic system during placenta development and also increase in fibrin resulting from blood stagnation in the lower limbs due to uterus enlargement [13, 14]. Toward late pregnancy, D-dimer levels are increased, with those in the third trimester exceeding normal values (< 0.5 µg/ml) in ≥ 90% of patients [12, 15–19]. However, in this study, D-dimer levels after cesarean section were found to significantly decrease to 7.5 (1.1–34.1) and 4.2 (0.02–31.4) µg/ml on postoperative days 1 and 6, respectively [10]. Subsequently, they returned to pre-pregnancy levels at around 6 weeks postpartum [20, 21]. Therefore, we performed CUS in 135 (26.5%) patients; however, none of the patients presented with a thrombus. Although the frequency of asymptomatic DVT after a cesarean section has been reported to be 3.9% [12], the frequency of preoperative asymptomatic DVT is likely to be low. These findings suggest that DVT is highly likely to occur during the perioperative period. Therefore, there is no considerable utility in preoperative DVT screening, while intraoperative and postoperative DVT prevention is crucial. Additionally, DVT visualization in the pelvis becomes challenging in the second half of pregnancy because of uterus enlargement. Since this increases the burden on laboratory technicians, it should be postoperatively performed to allow work efficiency and diagnostic accuracy [3, 22, 23]. However, future studies should determine the selection criteria for postoperative examinations.

Currently, the most predominant theory of HDP pathology is the two-step theory, that is, placental dysplasia (first step) at 10–18 weeks’ gestation and maternal vascular endothelial damage (second step) after 20 gestation weeks [24]. These damages neutralize angiogenic factors (placenta growth factor and vascular endothelial growth factor) and vascular endothelial damage, which causes high blood pressure and proteinuria. Several studies have reported elevated D-dimer levels in HDP (gestational hypertension) and preeclampsia [25, 26]. Furthermore, D-dimer levels may reflect disease severity [27]. This could be attributed to the exhibition of a hypercoagulable state in vascular endothelial damage. Moreover, the advanced fibrinolytic system increases D-dimer levels, which is a fibrin degradation product. Uttam et al. examined D-dimer levels in preeclampsia, severe preeclampsia, gestational diabetes mellitus, premature rupture of membranes, and preterm premature rupture of membranes. They observed elevated D-dimer levels in severe preeclampsia [18], which is consistent with our findings. This indicates that HDP is a risk factor for DVT that causes high D-dimer levels. In addition to the hypercoagulable state of pregnancy, HDP may have caused placental dysplasia and vascular endothelial damage given its characteristic pathology; moreover, the hypercoagulation/fibrinolytic system may have further progressed to yield high D-dimer levels.

Recent studies have strongly recommended developing postoperative anticoagulants and early ambulation for VTE prevention, which have decreased the VTE frequency. Specifically, the VTE frequency after a cesarean section was found to decrease by 40% compared with the frequency in the 1990s [28–30]. We postoperatively administered enoxaparin in 180 (36.2%) patients, with none of the patients showing clinical VTE.

### Clinical implications

To the best of our knowledge, this is the first study to demonstrate the low utility of the D-dimer cut-off value of 3.0 µg/ml in the third trimester as a DVT screening. Moreover, our findings suggest that CUS for asymptomatic thrombus before cesarean section may be of little significance.

### Research implications

There is a need for future clinical studies with continuous intervention to consistently collect data at the early phase of pregnancy, as well as during preoperative, postoperative, and postpartum periods. Accordingly, a multi-center study is currently being planned involving other medical institutions.

### Strengths and limitations

This study has several limitations. First, this was a single-center and small-scale study. Because most of the cases examined are low-risk DVT cases, it is important to require more extensive clinical data for high-risk cases. Second, we did not investigate temporal changes in coagulation factors. Since the blood coagulation ability easily changes during pregnancy, time-course measurements may be required. Third, we did not evaluate DVT within 4 post-delivery weeks using CUS. Therefore, the presence of asymptomatic DVT could not be excluded. In addition, our study has not examined the risk of anatomical malformations such as inferior vena cava atresia or May-Thurner Syndrome.

A strength of our study is that standardized CUS was performed in a single center. Moreover, we only included patients undergoing elective cesarean sections. By only including patients with a delivery period of 37–38 weeks, we minimized the bias among patients in the timing of D-dimer measurements, delivery period, and postpartum process.

## Conclusions

There may be low utility in screening for DVT using D-dimer levels in the third trimester. Further, prepartum asymptomatic DVT has a low frequency, indicating the low utility of CUS.

## Data Availability

All data generated or analyzed during this study are included in this published article.

## Abbreviations

BMI: Body mass index
CT: Computed tomography
CUS: Compression ultrasonography
DVT: Deep vein thrombosis
HDP: Hypertensive disorders of pregnancy
PVT: Pulmonary thromboembolism
VTE: Venous thromboembolism
